# Non-invasive imaging biomarkers of cellular injury and proliferation in nasopharyngeal carcinoma: insights from multiparametric MRI

**DOI:** 10.3389/fcell.2025.1684620

**Published:** 2025-11-07

**Authors:** Changjiang Zhao, Lisi Xie, Mengxi He, Xiong Xiong, Chengxin Yu, Yang Liu

**Affiliations:** 1 Department of Radiology, The First College of Clinical Medical Science, China Three Gorges University, Yichang Central People’s Hospital, Yichang, Hubei, China; 2 Institute of Medical Imaging, China Three Gorges University, Yichang, Hubei, China; 3 Department of Nuclear Medicine, Wuhan Children’s Hospital, Tongji Medical College, Huazhong University of Science and Technology, Wuhan, Hubei, China; 4 Department of Pathology, Yichang Central People’s Hospital and The First College of Clinical Medical Science, China Three Gorges University, Yichang, Hubei, China; 5 Department of Oncology, Yichang Central People’s Hospital and The First College of Clinical Medical Science, China Three Gorges University, Yichang, Hubei, China

**Keywords:** cellular injury, proliferation biomarker, intravoxel incoherent motion (IVIM), dynamic contrast-enhanced MRI (DCE-MRI), Ki-67, nasopharyngeal carcinoma

## Abstract

**Background:**

Early identification of therapeutic response and tumor proliferative status is essential in nasopharyngeal carcinoma (NPC). Multiparametric MRI-combining IVIM and DCE-provides quantitative biomarkers reflecting tissue diffusion, perfusion, and vascular permeability. We evaluated whether pre-treatment IVIM- and DCE-derived parameters predict short-term response to induction chemotherapy plus concurrent chemoradiotherapy and whether they correlate with tumor proliferation (Ki-67).

**Methods:**

In this prospective study (n = 48; January 2021–January 2023), IVIM parameters (D, D*, f) and DCE parameters (K^trans^, K_ep_, V_e_, V_p_) were quantified before treatment. Treatment response at 6 months was classified by RECIST 1.1 as complete response (CR) or non-CR. Ki-67 index was determined by immunohistochemistry (cutoff 50%). Group comparisons used t-tests or Mann–Whitney U tests; logistic regression identified independent predictors; ROC analysis evaluated diagnostic performance; Spearman correlation tested associations with Ki-67.

**Results:**

Pre-treatment D was lower in the CR group (0.82 ± 0.12 vs. 0.92 ± 0.11 ×10^-3^ mm^2^/s; *P* = 0.007). K^trans^ and K_ep_ were higher in CR (0.95 ± 0.34 vs. 0.30 ± 0.31 min^-1^, *P* = 0.014; 0.16 ± 0.09 vs. 0.11 ± 0.06 min^-1^, *P* = 0.025). D was an independent predictor (*P* = 0.008). The combined model (D + K^trans^ + K_ep_) yielded AUC = 0.834 (sensitivity 90.0%; specificity 61.4%). Ki-67 correlated negatively with D (r = −0.329, *P* = 0.022) and positively with V_p_ (r = 0.292, *P* = 0.044).

**Conclusion:**

Multiparametric MRI can noninvasively predict short-term response and reflect proliferative status in NPC. Integrating IVIM-derived D with DCE-derived K^trans^ and K_ep_ improves early prediction of treatment efficacy; V_p_ may serve as an imaging surrogate for proliferation.

## 1 Introduction

Nasopharyngeal carcinoma (NPC) is a malignant tumor of epithelial origin that develops from the mucosal lining of the nasopharynx (Chen et al., 2019). Its occurrence shows a pronounced geographic disparity, with particularly high incidence rates in Southern China, Southeast Asia, and parts of North Africa (Chen et al., 2019; You et al., 2023). Although modern treatment combining induction chemotherapy (IC) and concurrent chemoradiotherapy (CCRT) has improved clinical outcomes, patient responses remain heterogeneous (Chen et al., 2019; You et al., 2023; Zhou et al., 2020). Some individuals still demonstrate limited therapeutic benefit or experience early relapse (Liu et al., 2024). Rapid and accurate evaluation of treatment efficacy is therefore critical to guide therapeutic adjustments, reduce unnecessary adverse effects, and enhance long-term prognosis (Chen et al., 2019).

Therapeutic response in NPC reflects a balance between treatment-induced cytotoxicity and subsequent cellular repair (Chen et al., 2019; Luo, 2023). Conventional magnetic resonance imaging (MRI) primarily captures size changes, which occur later than the underlying microstructural and functional alterations. Functional imaging biomarkers that quantify early changes in diffusion, perfusion, and vascular permeability may thus provide earlier and more biologically meaningful insights into treatment response.

Multiparametric MRI offers a noninvasive approach to assess tumor microenvironmental characteristics. Intravoxel incoherent motion (IVIM) imaging distinguishes pure molecular diffusion from microvascular perfusion, yielding parameters such as the pure diffusion coefficient (D), pseudodiffusion coefficient (D*), and perfusion fraction (f), each sensitive to variations in cell density and microvascular status (Le Bihan, 2019; Iima, 2021; Le Bihan et al., 1988). Dynamic contrast–enhanced MRI (DCE-MRI) estimates vascular and tissue characteristics through metrics including the volume transfer constant (K^trans^), rate constant (K_ep_), extravascular extracellular space fractional volume (V_e_), and plasma volume fraction (V_p_) (Petralia et al., 2020; Gaustad et al., 2020; Cuenod and Balvay, 2013). These parameters collectively offer insight into microstructural damage, vascular compromise, and tissue remodeling following therapy (Petralia et al., 2020; Gaustad et al., 2020; Cuenod and Balvay, 2013).

Cell proliferation, indicated by the nuclear protein Ki-67, is a well-established prognostic marker in NPC and other malignancies (Shi et al., 2020; Menon et al., 2019; Li et al., 2021). However, biopsy-based assessment is limited by sampling variability and invasiveness (Shi et al., 2020; Menon et al., 2019). Imaging-based biomarkers correlated with proliferative activity could enable noninvasive, whole-tumor evaluation of biological response.

**FIGURE 1:**
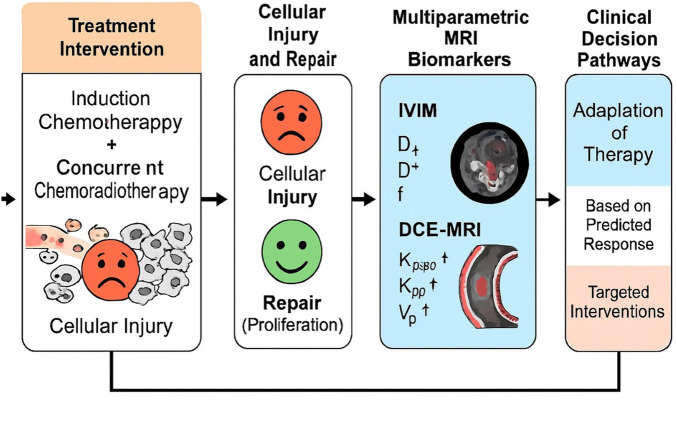
Study flowchart.

Based on these considerations, we hypothesized that quantitative parameters derived from pretreatment IVIM and DCE-MRI could serve as mechanistically informative biomarkers for predicting therapeutic response and tumor proliferation in NPC. Specifically, our objectives were: (i) to compare MRI-derived quantitative metrics between patients achieving complete versus incomplete responses to IC followed by CCRT; (ii) to determine the predictive accuracy of these parameters individually and in combination; and (iii) to investigate their correlation with Ki-67 expression, aiming to identify imaging-based indicators of proliferative status. By integrating advanced functional MRI with histopathological proliferation markers, this study seeks to link imaging phenotypes to underlying mechanisms of cellular injury and repair, ultimately supporting precision-tailored treatment strategies for NPC (Figure 1).

## 2 Materials and methods

### 2.1 Study population

#### 2.1.1 Ethical approval and study design

Approval for the research protocol was granted by the Ethics Committee of Yichang Central People’s Hospital (Ethics approval number: YCZXYY2021-005-01), adhering to the principles of the Declaration of Helsinki. Written informed consent was obtained from each participant prior to enrollment. This prospective observational study involved 48 patients newly diagnosed with NPC, confirmed histopathologically, who were treated at Yichang Central People’s Hospital between January 2021 and January 2023. The sample size was determined using G*Power software (version 3.1) (Faul et al., 2007), with an anticipated area under the curve (AUC) of 0.80 for the primary outcome, a power of 80%, and a significance level of 0.05. A total of 48 participants were enrolled, as this sample size meets the necessary statistical power requirements for the analysis.

#### 2.1.2 Eligibility criteria

Inclusion required: (i) pathologic confirmation of NPC via nasopharyngoscopy-guided biopsy; (ii) no previous oncologic treatment; (iii) a treatment plan involving IC followed by CCRT; and (iv) absence of contraindications to MRI, such as pacemaker implantation, severe claustrophobia, or cochlear implants. Patients were excluded if (i) image quality was degraded by motion or technical artifacts, or (ii) they failed to complete therapy due to health-related or financial constraints.

### 2.2 MRI acquisition protocol

All imaging was performed using a Philips 3.0T superconducting MRI scanner equipped with a head-and-neck coil. The protocol included axial T1-weighted (T1WI), T2-weighted (T2WI), fat-suppressed T2WI, and sagittal/coronal sequences, in addition to IVIM and DCE-MRI before initiation of treatment.

For IVIM, a two-dimensional spin-echo echo-planar sequence with selective excitation was employed, incorporating eight b-values (0, 10, 20, 50, 100, 150, 200, and 500 s/mm^2^). Parameters were: repetition time (TR) = 3000 ms, field of view (FOV) = 240 × 240 mm^2^, matrix = 256 × 129, slice thickness = 3 mm, and interslice gap = 0.3 mm.

For DCE-MRI, a 3D spoiled gradient echo (3D-SPGR) T1-weighted fat-suppressed sequence was used. T1 mapping was obtained with flip angles of 3°, 6°, 9°, 12°, and 15°, followed by dynamic acquisition at a fixed 12° angle. Gadodiamide (0.1 mmol/kg) was administered intravenously at 2 mL/s, chased by 20 mL saline. Twenty-six phases were captured, generating 260 dynamic images.

### 2.3 Treatment regimen

All patients received two cycles of IC with docetaxel (75 mg/m^2^) and nedaplatin (80 mg/m^2^) every 21 days. Two weeks later, intensity-modulated radiotherapy (IMRT) was initiated, delivering 70 Gy over 7 weeks (2 Gy per fraction, 5 fractions per week). Weekly nedaplatin was given CCRT.

### 2.4 Short-term treatment response assessment

Six months after completion of chemoradiotherapy, follow-up MRI was performed. Tumor response was assessed per response evaluation criteria in solid tumors (RECIST) v 1.1 (Nguyen et al., 2015):

- *Complete response (CR)*: total disappearance of target lesions;
- *Partial response (PR)*: ≥30% reduction in sum of target lesion diameters;
- *Stable disease (SD)*: neither PR nor progressive disease;
- *Progressive disease (PD)*: ≥20% increase in lesion size.

Patients in the CR category were classified as the *complete response group*, whereas those with PR, SD, or PD were placed in the *non-complete response group (non-* CR*)*.

### 2.5 Image postprocessing and quantitative analysis

IVIM-DWI: Diffusion data were converted via MRIcroGL and segmented manually in ITK-SNAP. Regions of interest (ROIs) were drawn on three consecutive axial slices, avoiding necrotic or cystic tissue. The D, D*, and f were calculated using a segmented biexponential model with Levenberg–Marquardt fitting (Sijtsema et al., 2021).

DCE-MRI: Data were analyzed with MR permeability software using the Tofts pharmacokinetic model and a population-based arterial input function (AIF). ROIs encompassed the largest tumor cross-section over five slices, excluding non-enhancing areas. The computed parameters included K^trans^, K_ep_, V_e_, and V_p_. Mean values were recorded for analysis (Khalifa et al., 2014).

### 2.6 Immunohistochemistry (IHC) and Ki-67 quantification

Formalin-fixed, paraffin-embedded NPC tissue blocks were sectioned at 4 μm. Slides were baked at 75 °C for 45 min, then dewaxed in xylene (three cycles, 5 min each) and rehydrated through graded ethanol. Antigen retrieval was performed in Ethylenediaminetetraacetic Acid (EDTA) buffer (pH 9.0) per manufacturer guidance. Ki-67 immunostaining followed a three-step protocol: incubation with primary monoclonal antibody, secondary antibody application, and 3,3′-Diaminobenzidine (DAB) chromogen development. Two experienced pathologists independently reviewed each case. The Ki-67 labeling index (LI) was calculated as the proportion of positively stained nuclei among 1,000 tumor cells in five high-power fields. A threshold of 50% distinguished low (≤50%) from high (>50%) expression groups.

### 2.7 Statistical analysis

Analyses were conducted in SPSS (version 16.0; Chicago, IL). The normality of continuous variables was assessed using the Kolmogorov–Smirnov test. Variables following a normal distribution were expressed as mean ± standard deviation (SD) and compared between groups using the independent-samples *t*-test. Non-normally distributed variables were presented as median (interquartile range, IQR) and compared using the Mann–Whitney *U* test. Categorical variables were summarized as frequencies and compared using the chi-square (*χ* ^*2*^) test or Fisher’s exact test as appropriate.

To evaluate the predictive performance of imaging parameters for treatment response to induction chemotherapy combined with concurrent chemoradiotherapy in nasopharyngeal carcinoma, univariate binary logistic regression analyses were first conducted to identify potential predictors associated with therapeutic efficacy. Variables with *P* < 0.10 in univariate analysis, as well as clinically relevant confounding factors (e.g., age, sex, and clinical stage), were subsequently entered into multivariate binary logistic regression models to calculate adjusted odds ratios (ORs) and 95% confidence intervals (CIs).

Given that multiple IVIM and DCE-MRI parameters were assessed, Bonferroni correction was applied to adjust the significance level (α) during multiple group comparisons and when constructing multiple logistic regression models, in order to control for the family-wise error rate. Finally, diagnostic models were developed based on independently significant predictors, and receiver operating characteristic (ROC) curves were generated to evaluate diagnostic performance by calculating the area under the curve (AUC). Comparisons between AUCs of different parameters or models were performed using DeLong’s test. All statistical analyses were two-tailed, and a Bonferroni-adjusted *P* < 0.05 was considered statistically significant.

## 3 Results

### 3.1 Patient demographics and clinical profile

The study included 48 patients diagnosed with NPC, categorized into two groups based on their therapeutic response at 6 months: complete response (CR, n = 28) and non-complete response (non-CR, n = 20). The baseline clinical characteristics of both groups are summarized in Table 1. No significant differences were observed between the groups in terms of age (*P* = 0.738), sex (*P* = 0.111), or clinical stage (*P* = 0.658).

**TABLE 1:** Baseline clinical characteristics of patients with nasopharyngeal carcinoma.

Characteristic	Total (n = 48)	CR (n = 28)	Non-CR (n = 20)	U/χ^2^	*P* Value
Age (years, mean ± SD)	52.9 ± 10.2	52.0 ± 11.7	53.6 ± 9.2	−0.335	0.738^a^
Sex				2.535	0.111^b^
Male	35	18	17		
Female	13	10	3		
Clinical stage				0.196	0.658^b^
III	21	13	8		
IV	27	15	12		

^a^ Mann-Whitney U test.

^b^ :χ2 test. CR, complete response; non-CR, non-complete response.

### 3.2 Pre-treatment IVIM parameter comparison

**FIGURE 2:**
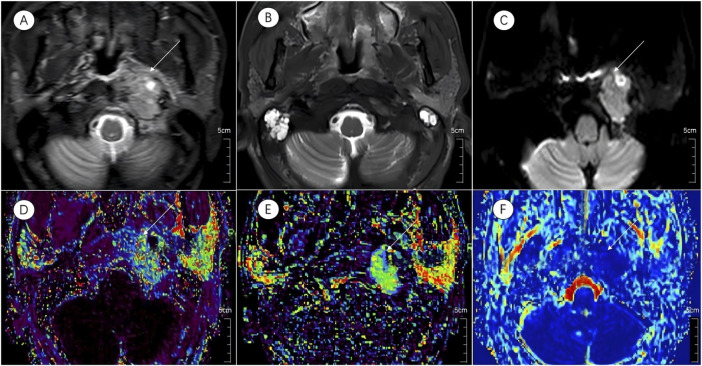
Multiparametric MR images of a representative patient from the complete response group. White arrows in the figure indicate lesions. **(A,B)** Axial T2-STIR images obtained before treatment and at the 6-month follow-up after induction chemotherapy (IC) followed by concurrent chemoradiotherapy (CCRT). Image **(B)** demonstrates near-complete resolution of the primary lesion.**(C)** IVIM map of the lesion before treatment. **(D–F)** Parametric maps of *K* ^*trans*^, *K*_*ep*_, and *D* obtained before treatment.

**FIGURE 3:**
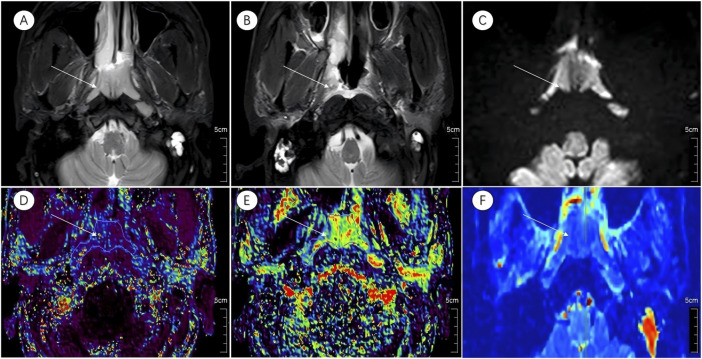
Multiparametric MR images of a representative patient from the incomplete response group. White arrows in the figure indicate lesions. **(A,B)** Axial T2-STIR images acquired before treatment and at the 6-month follow-up after induction chemotherapy (IC) followed by concurrent chemoradiotherapy (CCRT). Image **(B)** shows partial residual tumor following treatment. **(C)** IVIM map of the lesion before treatment.**(D–F)** Parametric maps of *K* ^*trans*^, *K*_*ep*_, and *D* obtained before treatment.

Prior to treatment initiation, the CR group exhibited a markedly lower D than the non-CR group (0.82 ± 0.12 × 10^−3^ mm^2^/s vs. 0.92 ± 0.11 × 10^−3^ mm^2^/s; t = −2.824, *P* = 0.007). No significant intergroup differences were detected in the D* or f (*P* > 0.05 for both). Graphical and tabular data are displayed in Figures 2, 3 and Table 2.

**TABLE 2:** Comparison of pretreatment IVIM and DCE-MRI parameters between CR and non-CR groups.

Parameter	CR(n = 28)	Non-CR(n = 20)	t/Z	P Value
D (×10^−3^ mm^2^/s)	0.82 ± 0.12	0.92 ± 0.11	−2.824	0.007*
D*(×10^−3^ mm^2^/s)	52.80 ± 13.31	50.44 ± 18.88	0.516	0.609
f (%)	0.10 ± 0.05	0.12 ± 0.06	−1.024	0.311
K^trans^ (min^-1^)	0.95 ± 0.34	0.30 ± 0.31	2.557	0.014*
K_ep_ (min^-1^)	0.16 ± 0.09	0.11 ± 0.06	2.322	0.025*
V_e_	0.79 ± 0.57	1.01 ± 1.02	−0.711	0.477
V_p_	0.46 ± 0.17	0.49 ± 0.16	−0.569	0.572

*P < 0.05. CR, complete response; non-CR, non-complete response; D, pure diffusion coefficient; D*, pseudodiffusion coefficient; f, perfusion fraction; K^trans^, volume transfer constant; K_ep_, rate constant; V_e_, extravascular extracellular space fractional volume; V_p,_ plasma volume fraction.

### 3.3 Pre-treatment DCE-MRI parameter comparison

Analysis of DCE-MRI parameters revealed significantly elevated K^trans^ and K_ep_ values in the CR group relative to the non-CR group (K^trans^: 0.95 ± 0.34 min^-1^ vs. 0.30 ± 0.31 min^−1^, t = 2.557, *P* = 0.014; K_ep_: 0.16 ± 0.09 min^−1^ vs. 0.11 ± 0.06 min^−1^, t = 2.322, *P* = 0.025). V_e_ and V_p_ showed no significant differences between the two cohorts (*P* = 0.477 and *P* = 0.572, respectively). Detailed outcomes are shown in Figures 2, 3 and Table 2.

### 3.4 Logistic regression analysis

Binary logistic regression was applied to identify imaging-derived predictors of complete response. Among all pre-treatment metrics, the D value emerged as the only independent predictor (OR = 2.299; 95% CI: 1.625–4.897; p = 0.008). Neither K^trans^ nor K_ep_ reached statistical significance (*P* = 0.094 and *P* = 0.193). These results are summarized in Table 3.

**TABLE 3:** Binary logistic regression analysis of pretreatment D, K^trans^, and K_e**p**_ values for short-term therapeutic response.

Parameter	OR	95% CI	*P* Value
D (×10^−3^ mm^2^/s)	2.299	1.625–4.897	0.008*
K^trans^ (min^−1^)	0.979	0.955–1.004	0.094
K_ep_ (min^−1^)	0.992	0.981–1.004	0.193

*P < 0.05. OR, odds ratio; 95% CI, 95% confidence interval; D, pure diffusion coefficient; K^trans^, volume transfer constant; K_ep_, rate constant.

### 3.5 Diagnostic performance assessment

#### 3.5.1 Diagnostic performance of pre-treatment parameters

**FIGURE 4:**
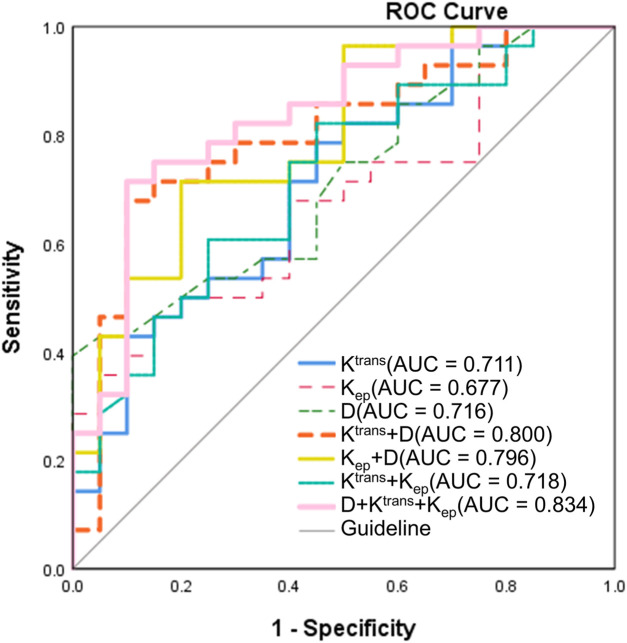
ROC curves for predicting short-term treatment response in NPC. ROC analysis was performed to evaluate the diagnostic performance of pre-treatment MRI-derived parameters. Among single-parameter models, the diffusion coefficient D exhibited the highest sensitivity (100%) but relatively low specificity (39.3%), yielding an AUC of 0.716 (95% CI: 0.573–0.859, *P* = 0.011). K^trans^ (AUC = 0.711) and K_ep_ (AUC = 0.677) showed lower diagnostic efficiencies. Combining parameters markedly enhanced predictive accuracy: The K^trans^ + D and K_ep_ + D models achieved AUCs of 0.800 and 0.796, respectively, while the three-parameter model (D + K^trans^ + K_ep_) demonstrated the best overall performance (AUC = 0.834; 95% CI: 0.716–0.952; *P* < 0.001) with 90% sensitivity and 61.4% specificity. (ROC, receiver operating characteristic; NPC, nasopharyngeal carcinoma; AUC, area under the curve; CI, confidence interval; D, pure diffusion coefficient; Ktrans, volume transfer constant; Kep, rate constant).

ROC curve analyses were conducted to evaluate the predictive value of pre-treatment MRI parameters for short-term therapeutic response (Figure 4; Table 4). The diagnostic performance metrics—including optimal threshold, sensitivity, specificity, AUC, and corresponding *P* values—were summarized accordingly. Among the single-parameter models, the D demonstrated the highest sensitivity (100%) at a threshold of 0.755 × 10^−3^ mm^2^/s, although its specificity was relatively low (39.3%), yielding an AUC of 0.716 (95% CI: 0.573–0.859, *P* = 0.011). The K^trans^ (threshold: 0.637 min^−1^) showed a sensitivity of 78.6% and specificity of 33.6%, with an AUC of 0.711 (95% CI: 0.563–0.858, *P* = 0.014). In contrast, the K_ep_ (threshold: 0.158 min^−1^) exhibited the lowest diagnostic efficiency (sensitivity: 46.4%; specificity: 31.4%; AUC = 0.677; 95% CI: 0.526–0.828; *P* = 0.038). Combining parameters markedly improved diagnostic performance. The K^trans^ + D model achieved an AUC of 0.800 (95% CI: 0.670–0.930, *P* < 0.001), with a sensitivity of 90% and specificity of 57.9%. The K_ep_ + D model yielded comparable performance (AUC = 0.796; 95% CI: 0.669–0.923; *P* = 0.001). Although the K^trans^ + K_ep_ combination provided only modest improvement (AUC = 0.718), the three-parameter model D + K^trans^ + K_ep_ demonstrated the best overall diagnostic efficacy, achieving an AUC of 0.834 (95% CI: 0.716–0.952, *P* < 0.001), with 90% sensitivity and 61.4% specificity. These results identify the three-parameter combination as the optimal model for predicting short-term treatment response.

**TABLE 4:** Diagnostic performance of pretreatment parameters for discriminating short-term therapeutic response.

Parameter	Threshold	Sensitivity (%)	Specificity (%)	AUC (95% CI)	*P* Value
D(×10^−3^ mm^2^/s)	0.755	100	39.3	0.716(0.573,0.859)	0.011*
K^trans^ (min^−1^)	0.637	78.6	33.6	0.711(0.563,0.858)	0.014*
K_ep_ (min^−1^)	0.158	46.4	31.4	0.677(0.526,0.828)	0.038*
K^trans^ + D	0.302	90	57.9	0.800(0.670,0.930)	<0.001*
K_ep_ + D	0.400	80	51.4	0.796(0.669,0.923)	0.001*
K^trans^ + K_ep_	0.534	55	37.1	0.718(0.572,0.864)	0.011*
D + K^trans^ + K_ep_	0.363	90	61.4	0.834(0.716,0.952)	<0.001*

*P < 0.05. AUC, area under the curve; 95% CI, 95% confidence interval; D, pure diffusion coefficient; K^trans^, volume transfer constant; K_ep_, rate constant.

#### 3.5.2 Comparison of diagnostic performance by DeLong’s test

Pairwise comparisons of AUCs were performed using DeLong’s test to determine the statistical significance of performance differences (Table 5). Among individual parameters, K^trans^ demonstrated a significantly higher AUC than both K_ep_ (AUC difference = 0.034, *P* = 0.044) and D (AUC difference = −0.005, *P* = 0.023). A significant difference was also observed between K_ep_ and D (*P* = 0.032). Importantly, the three-parameter model D + K^trans^ + K_ep_ achieved significantly higher AUCs than any single parameter, including K^trans^ (AUC difference = −0.123, *P* = 0.039), K_ep_ (AUC difference = −0.157, *P* = 0.041), and D (AUC difference = −0.118, *P* = 0.046). However, comparisons among combined models revealed no statistically significant differences between D + K^trans^ + K_ep_ and either K^trans^ + D (AUC difference = −0.034, *P* = 0.198) or K_ep_ + D (AUC difference = −0.038, *P* = 0.343). Likewise, K^trans^ + D and K_ep_ + D did not differ significantly (AUC difference = 0.004, *P* = 0.950).

**TABLE 5:** Comparison of diagnostic performance by Delong’s tes**t**.

Parameter	Z	AUC difference	(95% CI)	*P* Value
K^trans^ VS. K_ep_	0.462	0.034	−0.110–0.178	0.044^*^
K^trans^ VS. D	−0.047	−0.005	−0.229–0.219	0.023^*^
K^trans^ VS. K^trans^ + D	−1.361	−0.089	−0.218–0.039	0.174
K^trans^ VS. K_ep_ + D	−0.931	−0.086	−0.266–0.095	0.352
K^trans^ VS. K^trans^ + K_ep_	−0.269	−0.008	−0.067–0.050	0.788
K^trans^ VS. D + K^trans^ + K_ep_	−1.866	−0.123	−0.251–0.005	0.039^*^
K_ep_ VS. D	−0.343	−0.039	−0.264–0.185	0.032^*^
K_ep_ VS. K^trans^ + D	−1.334	−0.123	−0.304–0.058	0.182
K_ep_ VS. K_ep_ + D	−1.597	−0.120	−0.267–0.027	0.110
K_ep_ VS. K^trans^ + K_ep_	−0.801	−0.042	−0.145–0.061	0.423
K_ep_ VS. D + K^trans^ + K_ep_	−2.044	−0.157	−0.308∼-0.006	0.041^*^
D VS. K^trans^ + D	−1.332	−0.084	−0.207–0.040	0.183
D VS. K_ep_ + D	−1.451	−0.080	−0.189–0.028	0.147
D VS. K^trans^ + K_ep_	−0.024	−0.003	−0.223–0.218	0.981
D VS. D + K^trans^ + K_ep_	−1.841	−0.118	−0.243–0.008	0.046^*^
K^trans^ + D VS. K_ep_ + D	0.063	0.004	−0.107–0.114	0.950
K^trans^ + D VS. K^trans^ + K_ep_	1.190	0.081	−0.053–0.215	0.234
K^trans^ + D VS. D + K^trans^ + K_ep_	−1.287	−0.034	−0.086–0.018	0.198
K_ep_ + D VS. K^trans^ + K_ep_	0.961	0.078	−0.081–0.236	0.337
K_ep_ + D VS. D + K^trans^ + K_ep_	−0.948	−0.038	−0.115–0.040	0.343
K^trans^ + K_ep_ VS. D + K^trans^ + K_ep_	−1.943	−0.115	−0.231–0.001	0.052

*P < 0.05. AUC, area under the curve; 95% CI, 95% confidence interval; D, pure diffusion coefficient; K^trans^, volume transfer constant; K_ep_, rate constant.

Collectively, both ROC and DeLong’s analyses confirmed that multiparametric models—particularly D + K^trans^ + K_ep_—substantially enhance predictive accuracy compared with individual parameters, underscoring their potential clinical utility in early response assessment.

### 3.6 Correlation of imaging biomarkers with Ki-67 expression

**FIGURE 5:**
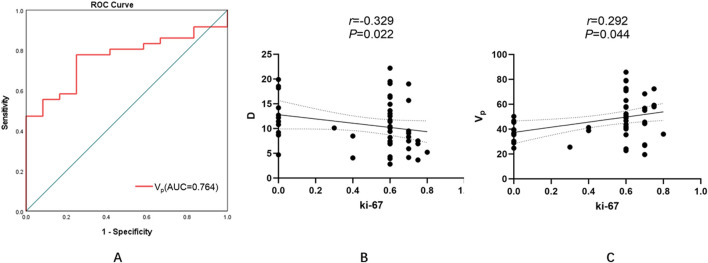
Diagnostic performance and correlations of MRI-derived parameters with tumor proliferative activity (Ki-67 index). **(A)** ROC curve analysis of the V_p_ for predicting high Ki-67 expression. The optimal threshold of 0.399 yielded an AUC of 0.764, with a sensitivity of 77.8% and specificity of 52.8%. **(B)** A moderate negative correlation between D and Ki-67 was observed (r = −0.329, P = 0.022), indicating that lower diffusivity is associated with increased proliferative activity. **(C)** A positive correlation between Vp and Ki-67 (r = 0.292, P = 0.044), suggesting that higher perfusion correlates with accelerated cellular turnover. (ROC, receiv*er operating characteristic; AUC, area under the curve; D, pure diffusion coefficient; Vp, plasma volume fraction)*.

Immunohistochemical evaluation identified 32 patients (66.7%) with high Ki-67 expression (≥50%) and 16 patients (33.3%) with low expression (<50%). Spearman’s rank correlation revealed:

- A moderate inverse relationship between D and Ki-67 (r = −0.329, *P* = 0.022), indicating that reduced diffusivity corresponds to increased proliferative activity (Figure 5B; Table 6).
- A positive association between V_p_ and Ki-67 (r = 0.292, *P* = 0.044), suggesting that higher perfusion is linked with accelerated cellular turnover (Figure 5C; Table 6).
- No significant associations for D*, f, K^trans^, K_ep_, or V_e_ (all *P* > 0.05) (Table 6).

**TABLE 6:** Correlation between pretreatment MRI parameters and Ki-67 index.

Parameter	r	*P* Value
D (×10^−3^ mm^2^/s)	−0.329	0.022*
D* (×10^−3^ mm^2^/s)	0.129	0.382
f (%)	0.213	0.147
K^trans^ (min^−1^)	0.034	0.816
K_ep_ (min^−1^)	−0.023	0.878
V_e_	0.013	0.931
V_p_	0.292	0.044*

*P < 0.05. r, correlation coefficient; D, pure diffusion coefficient; D*, pseudodiffusion coefficient; f, perfusion fraction; K^trans^, volume transfer constant; K_ep_, rate constant; V_e_, extravascular extracellular space fractional volume; V_p,_ plasma volume fraction.

**FIGURE 6:**
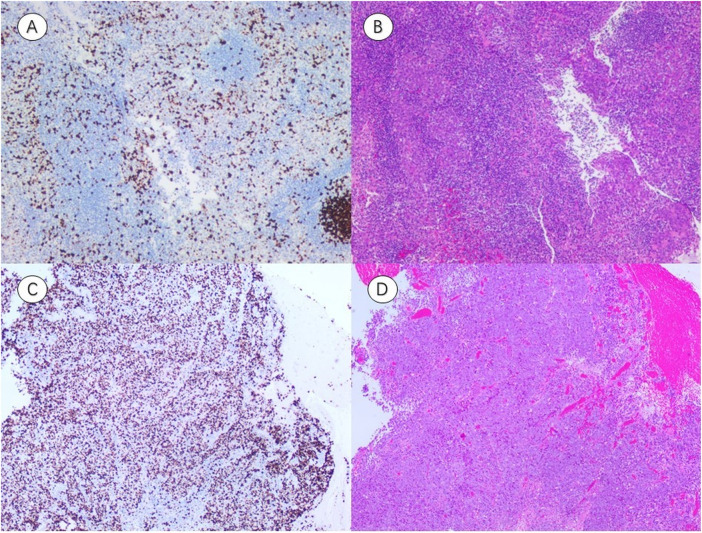
Representative immunohistochemistry (IHC) and hematoxylin–eosin (HE) staining of NPC tissues with different Ki-67 expression levels. **(A,B)** IHC image Ki-67(LI ≈ 30%) and corresponding HE-stained section from a patient with low Ki-67 expression (A threshold of 50% distinguished low (≤50%) from high (>50%) expression groups). **(C,D)** IHC image Ki-67(LI ≈ 80%) and corresponding HE-stained section from a patient with high Ki-67 expression (A threshold of 50% distinguished low (≤50%) from high (>50%) expression groups). (IHC, representative immunohistochemistry; HE, hematoxylin–eosin; LI, labeling index).

These findings highlight D and V_p_ as potential noninvasive surrogates for tumor proliferation and injury. Details are shown in Figures 5, 6 and Table 6.

### 3.7 Subgroup analysis by Ki-67 status

Patients with low Ki-67 expression demonstrated significantly lower V_p_ values than those with high expression (Z = −2.714, *P* = 0.007). No other parameter—including D, D*, f, K^trans^, K_ep_, or V_e_—showed statistically significant differences between the subgroups (Z = −1.191, *P* = 0.234; Z = −0.857, *P* = 0.391; Z = −1.762, *P* = 0.078; Z = −0.238, *P* = 0.812; Z = −0.571, *P* = 0.568; Z = −0.929, *P* = 0.353, respectively) (Table 7). For V_p_, the ROC curve analysis yielded an AUC of 0.764, with an optimal threshold of 0.399, achieving a sensitivity of 77.8% and specificity of 52.8% (Figure 5A; Table 8).

**TABLE 7:** Comparison of pretreatment parameters between low and high Ki-67 index expression group**s**.

Parameter	Low expression	High expression	Z	*P* Value
D (×10^−3^ mm^2^/s)	0.90 ± 0.08	0.85 ± 0.14	−1.191	0.234
D*(×10^−3^ mm^2^/s)	56.02 ± 14.53	50.44 ± 16.06	−0.857	0.391
f (%)	0.13 ± 0.04	0.10 ± 0.05	−1.762	0.078
K^trans^ (min^−1^)	0.83 ± 0.34	0.85 ± 0.35	−0.238	0.812
K_ep_ (min^−1^)	0.15 ± 0.08	0.13 ± 0.09	−0.571	0.568
V_e_	0.66 ± 0.35	0.96 ± 0.88	−0.929	0.353
V_p_	0.37 ± 0.08	0.51 ± 0.17	−2.714	0.007*

*P < 0.05. D, pure diffusion coefficient; D*, pseudodiffusion coefficient; f, perfusion fraction; K^trans^, volume transfer constant; K_ep_, rate constant; V_e_, extravascular extracellular space fractional volume; V_p,_ plasma volume fraction.

**TABLE 8:** Diagnostic performance of pretreatment V_**p**_ value in nasopharyngeal carcinoma high and low Ki-67 index expression group**s**.

Parameter	Threshold	Sensitivity (%)	Specificity (%)	AUC (95% CI)	*P* Value
V_p_	0.399	77.8	75.0	0.764(0.629,0.898)	0.007*

*P < 0.05. AUC, area under the curve; 95% CI, 95% confidence interval; V_p,_ plasma volume fraction.

## 4 Discussion

In this prospective analysis, multiparametric MRI parameters were evaluated for their ability to predict early treatment response and provide biological insight into tumor proliferation in NPC. A lower pretreatment D value, combined with higher K^trans^ and K_ep_, was significantly associated with CR after IC followed by CCRT. Among these, D was the sole independent predictor in multivariate analysis, while a combined model incorporating D, K^trans^, and K_ep_ achieved the highest predictive accuracy. Additionally, Ki-67 expression demonstrated a negative correlation with D and a positive correlation with V_p_, suggesting an association between vascular characteristics and proliferative potential.

### 4.1 Mechanistic interpretation

From a pathophysiological standpoint, cytotoxic chemotherapy and radiotherapy inflict damage through DNA disruption, oxidative stress, and vascular injury, ultimately impacting both tumor cellularity and microvascular integrity (Herrmann, 2020; Ping et al., 2020). Lower D values in CR patients may reflect higher baseline tumor cell density and restricted water diffusivity—features that could indicate a predominance of radiosensitive cells susceptible to irreversible injury. Elevated K^trans^ and K_ep_ suggest increased vascular permeability and perfusion, which may enhance delivery of cytotoxic drugs, promoting more effective tumor clearance.

The observed positive relationship between V_p_ and Ki-67 underscores the role of vascular architecture in sustaining rapid tumor cell proliferation. High V_p_ values in highly proliferative tumors may reflect a vascular network capable of meeting the metabolic demands of aggressive growth, reinforcing the concept that vascular remodeling is central to both the injury response and repair phases of tumor biology (Lidonnici et al., 2022). Multiparametric MRI thus provides not only quantitative biomarkers of these processes but also a direct translational link between imaging phenotypes and histopathological measures of proliferation.

### 4.2 Context within the literature

Over the past decade, consistent evidence has demonstrated that (i) diffusion-related parameters, such as those derived from DKI and IVIM-D, are sensitive indicators for early treatment response prediction; (ii) DCE-derived parameters (K^trans^ and K_ep_) reflect tissue perfusion and permeability, enabling the monitoring and prediction of therapeutic efficacy and correlating with biological markers such as Ki-67; and (iii) multiparametric, multimodal, or radiomics-based approaches generally outperform single-parameter models. For further details, refer to Table 9. Based on this rationale, our study integrates IVIM-D with DCE-derived K^trans^ and K_ep_ to enhance discrimination of short-term therapeutic response (AUC = 0.834). Moreover, the observed physiological associations with tumor proliferation (negative correlation of D and positive correlation of V_p_ with Ki-67) provide additional mechanistic insight. Together, these findings align with current evidence and offer quantifiable performance metrics, achieving a favorable balance between interpretability and clinical applicability with promising potential for broader implementation.

**TABLE 9:** Literature comparison.

Study author/year (journal)	Imaging technique	Main findings	Advantages	Limitations	Comparison with present study
Wang et al., 2023 (Clinical Radiology) (Wang et al., 2023)	MRI Radiomics (multi-sequence)	Radiomics model predicted IC response with internal validation.	Non-invasive; multi-feature model	Low interpretability; reproducibility challenges	Radiomics uses high-dimensional features; present study emphasizes physiologic interpretability with D/K^trans^/K_ep_.
Zhao et al., 2022 (Frontiers in Oncology) (Zhao et al., 2022)	Functional MRI (DWI/DKI)	Demonstrated diffusion-based parameters as early predictors of IC response.	Systematic comparison; clinical relevance	Technique variability; lack of external validation	Reinforces D as a sensitive marker; present study further improves prediction by integrating DCE parameters.
Zhao et al., 2021 (Cancer Imaging) (Zhao et al., 2021)	Multiparametric functional MRI (IVIM/DKI/DCE)	Evaluated multiple pre-treatment MRI parameters predicting IC response; multiparametric models improved discrimination.	Head-and-neck cohort; direct modality comparison	Single-center; small sample	Supports the superiority of multimodal integration; consistent with combining D + K^trans^ + K_ep_ achieving high AUC.
Wu et al., 2021 (Frontiers in Oncology) (Wu et al., 2021)	3D-pCASL and IVIM	3D-pCASL better distinguished high vs. low Ki-67 than IVIM; BFmax best marker.	Direct link with proliferation index	Sequence availability limited	Our findings complement with D negatively and Vp positively correlated with Ki-67.
Huang et al., 2021 (Radiotherapy & Oncology) (Huang et al., 2021)	DCE-MRI (QTM)	DCE parameters correlated with HIF-1α, EGFR, and Ki-67; indicating biological relevance.	Bridges imaging and molecular biology	Complex analysis; needs validation	Supports biological plausibility of K^trans^/K_ep_ association; our study validates predictive power and fusion benefit.
Tu et al., 2019 (AJNR) (Tu et al., 2019)	DKI histogram analysis	Histogram metrics predicted NAC response in NPC.	Simple to implement; clinically interpretable	Dependent on consistent acquisition	Both highlight microstructural complexity; present study’s fusion model shows improved AUC with DCE metrics.
Qin et al., 2018 (Medicine) (Qin et al., 2018)	IVIM-based texture analysis	GLCM texture features predicted CRT early response.	Non-invasive; related to diffusion metrics	Texture sensitive to protocol differences	Both address diffusion dimension; this study focuses on quantitative physiologic parameters with clinical thresholds.

### 4.3 Clinical and translational implications

Mechanism-focused imaging strategies may offer practical clinical benefits, including:

i. enabling early treatment modification-either intensification or de-escalation-based on predicted therapeutic response;
ii. identifying tumors with high proliferative potential, which may be candidates for anti-angiogenic or cell cycle-targeted therapy.

## 5 Study limitations and future directions

This study has several limitations. First, the relatively small, single-center cohort may restrict the generalizability of the findings. Although the sample size met the statistical requirement for detecting large effect sizes, it may not fully capture inter-patient heterogeneity in tumor biology and treatment response. Second, the use of a uniform treatment regimen (IC + CCRT) ensured methodological consistency but may not represent the diversity of real-world clinical practice. Third, although logistic regression models were constructed with a limited number of predictors to minimize overfitting, potential model instability cannot be completely ruled out due to the moderate sample size. Finally, the cross-sectional design limited our ability to infer causal relationships between imaging parameters and tumor proliferation (Ki-67).

Future studies should validate these findings in larger, multi-center cohorts and incorporate external datasets to enhance model robustness and reproducibility. Integration of advanced radiomics and machine-learning approaches may further improve the predictive performance and interpretability of multiparametric MRI. Additionally, longitudinal imaging combined with molecular or histopathological markers could help elucidate the dynamic interplay between tumor diffusion, perfusion, and proliferative activity. Ultimately, these efforts may support the development of individualized treatment strategies, allowing early therapy adaptation based on imaging-derived biomarkers.

## Data Availability

The original contributions presented in the study are included in the article/supplementary material, further inquiries can be directed to the corresponding authors.
